# Concordance of Peripheral Blood and Bone Marrow Next-Generation Sequencing in Hematologic Neoplasms

**DOI:** 10.1155/2022/8091746

**Published:** 2022-03-26

**Authors:** Chayanit Jumniensuk, Alexander Nobori, Thomas Lee, T. Niroshi Senaratne, Dinesh Rao, Sheeja Pullarkat

**Affiliations:** ^1^Department of Pathology, Phramongkutkloa Hospital and College of Medicine, Army Institute of Pathology, Bangkok, Thailand; ^2^Department of Pathology and Laboratory Medicine, University of California at Los Angeles, Ronald Reagan UCLA Medical Center, Los Angeles, CA 90095, USA

## Abstract

**Objective:**

Mutational analysis by next-generation sequencing (NGS) obtained by peripheral blood NGS has been of clinical interest to use as a minimally invasive screening tool. Our study evaluates the correlation between NGS results on peripheral blood and bone marrow in hematolymphoid disease.

**Method:**

We evaluated patients who had NGS for presumed hematologic malignancy performed on peripheral blood and bone marrow within a 1-year interval of each other. We excluded cases in which chemotherapy or bone marrow transplant occurred in the interval between the two tests. The concordance across peripheral blood and bone marrow NGS results was assessed by kappa coefficient analysis.

**Results:**

A total of 163 patients were studied. Concordance of peripheral blood and bone marrow NGS found in 150 patients (92.0%) with a kappa coefficient of 0.794 (kappa standard error 0.054) and *P* value for testing kappa <0.0001. Myeloid neoplasms showed concordant results in 77/78 cases (98.7%) with a kappa coefficient of 0.916. Lymphoid neoplasms showed concordant results in 26/31 cases (83.9%) with a kappa coefficient of 0.599. Nonneoplastic cases showed concordant results in 47/54 cases (87.0%) with a kappa coefficient of 0.743.

**Conclusion:**

Peripheral blood NGS is a reliable tool for mutational analysis and provides a less invasive method for screening and monitoring of the molecular profile.

## 1. Introduction

The diagnosis of hematolymphoid neoplasms requires the integration of multiple sources of patient data, including the clinical presentation, pathologic features such as morphology and immunophenotype from biopsy material, cytogenetics, and molecular characteristics [1]. While the relative importance of each parameter varies by disease, no single modality alone is sufficient to diagnose any hematolymphoid disease and all data must be integrated to form a diagnosis [1]. Genetic data are one of the critical factors in the diagnosis of some lymphoid and many myeloid neoplasms. In addition to their role in diagnosis, molecular characteristics can also provide prognostic and therapeutic information [1].

Mutational analysis by next-generation sequencing (NGS) is increasingly used in clinical practice when evaluating hematologic diseases [2]. However, the overall clinical significance of these NGS results have been a challenge to incorporate in clinical practice [2–4]. Currently, bone marrow aspiration and biopsy remain necessary for the initial diagnosis of neoplastic processes involving bone marrow [5]. Mutational analysis obtained by peripheral blood NGS has been of clinical interest to use as a screening tool due to the less invasive nature of this test [6].

Peripheral blood NGS has been described to be a reliable tool in screening for myeloid neoplasms in patients presenting with cytopenia, with a reported negative predictive value of 95% [6]. Conversely, the presence of a pathogenic mutation modestly predicted the presence of a myeloid neoplasm, confirmed by subsequent bone marrow biopsy (positive predictive value of 58%) [6].

To date, the concordance between NGS performed on peripheral blood and bone marrow in the evaluation of hematolymphoid malignancy overall has not been well studied. We sought to evaluate the relationship between NGS performed on peripheral blood and bone marrow specimens in the setting of the diagnosis of hematolymphoid disease. We also sought to determine the relative sensitivity and specificity of NGS on peripheral blood and bone marrow in the diagnosis of hematolymphoid disease.

## 2. Methods

**Figure 1:**
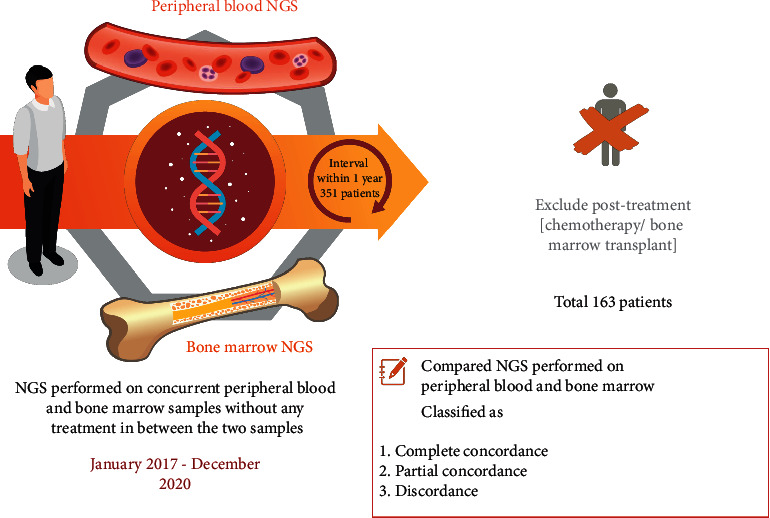
Diagram showing the selection of the study population.

After institutional review board approval, we retrospectively identified patients with peripheral blood NGS performed from January 1^st^, 2017 through December 31^st^, 2020. A total of 2403 patients were identified. Of these, 368 patients underwent NGS evaluation of a bone marrow specimen. We subsequently excluded patients in whom the interval between bone marrow and peripheral blood NGS was longer than 1 year, narrowing the study population to 351 patients. The patients' medical records were reviewed to identify the diagnosis, treatment history, and bone marrow biopsy result. Patients who underwent chemotherapy or bone marrow transplant were subsequently excluded, which included 162 patients who received chemotherapy and 26 patients who received bone marrow transplant, narrowing the study population to 163 patients (Figure 1).

We recorded NGS results and compared NGS performed on peripheral blood and bone marrow using the same gene panel. Cases were then classified as complete concordance, partial concordance, and discordance. Complete concordance was defined as the scenario in which bone marrow and peripheral blood NGS show an identical set of abnormal genes or genes. Partial concordance was defined as the scenario in which the two modalities identified some but not all of the same abnormal genes. Discordance was defined as the scenario in which there was no overlap between the genes detected in bone marrow and peripheral blood, either due to the detection of entirely different genes in the two modalities or the detection of abnormal genes in one modality but no detection in the other.

Statistical analysis was performed with descriptive statistics for demographic data. The concordance across peripheral blood and bone marrow NGS was assessed by the kappa coefficient. The kappa coefficient (*κ*) values indicated the strength of agreement based on Altman (1991) [7] as follows: poor *κ* ≤ 0.20, fair *κ* = 0.21–0.40, moderate *κ* = 0.41–0.60, good *κ* = 0.61–0.80, and very good *κ* = 0.81–1.00.

NGS testing was performed using the Illumina TruSight sequencing panel using a 54-gene comprehensive panel covering the most clinically significant mutations reported in hematologic malignancy. The genes included *ABL1*, *ASXL1*, *ATRX*, *BCOR*, *BCORL1*, *BRAF*, *CALR*, *CBL*, *CBLB*, *CBLC*, *CDKN2A*, *CEBPA*, *CSF3R*, *CUX1*, *DNMT3A*, *ETV6*, *EZH2*, *FBXW7*, *FLT3*, *GATA1*, *GATA2*, *GNAS*, *HRAS*, *IDH1*, *IDH2*, *IKZF1*, *JAK2*, *JAK3*, *KDM6A*, *KIT*, KMT2A, *KRAS*, *MPL*, *MYD88*, *NOTCH1*, *NPM1*, *NRAS*, *PDGFRA*, *PHF6*, *PTEN*, *PTPN11*, *RAD21*, *RUNX1*, *SETBP1*, *SF3B1*, *SMC1A*, *SMC3*, *SRSF2*, *STAG2*, *TET2*, *TP53*, *U2AF1*, *WT1*, and *ZRSR2*. For lymphoid malignancies an 18 gene panel included *BCOR*, *BRAF*, *CDKN2A*, *DNMT3A*, *EZH2*, *FBXW7*, *IDH1*, *IDH2*, *JAK2*, *JAK3*, *KIT*, *KRAS*, *MYD88*, *NOTCH1*, *NRAS*, *SF3B1*, *TET2*, and *TP53*.

## 3. Results

**Figure 2:**
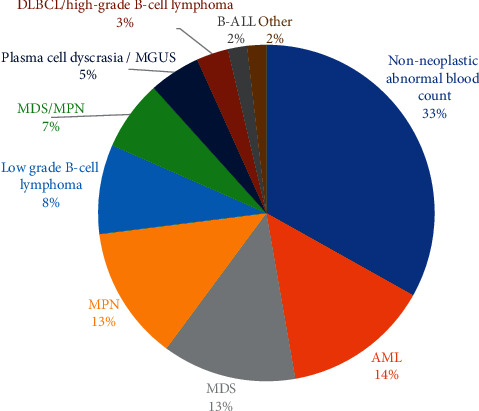
Distribution of diagnoses in the study population. MDS, myelodysplastic syndrome; AML, acute myeloid leukemia; MPN, myeloproliferative neoplasm; MGUS, monoclonal gammopathy of uncertain significance; DLBCL, diffuse large B-cell lymphoma; BALL, B-acute lymphoblastic leukemia.

One third of the patients within the study population were those who presented with an abnormal complete blood count (CBC) but whose subsequent bone marrow evaluation was not diagnostic for hematolymphoid disease. This group included 54 cases (33.1%). The CBC abnormalities seen included cytopenia(s), polycythemia, leukocytosis, thrombocytosis, and eosinophilia. Associated conditions included iron deficiency anemia, immune thrombocytopenia, reactive thrombocytosis, and idiopathic eosinophilia (Figure 2). Myeloid neoplasms consist of acute myeloid leukemia (AML) (23 cases, 14.1%), myelodysplastic syndrome (MDS) (21 cases, 12.9%), myeloproliferative neoplasm (MPN) (21 cases, 12.9%), myelodysplastic/myeloproliferative neoplasm (MDS/MPN) (11 cases, 6.7%), and mastocytosis (1 case, 0.6%).

31 cases (19%) were lymphoid neoplasms, including B-lymphoblastic leukemia (BALL) (3 cases, 2%), chronic lymphocytic leukemia (CLL) (5 cases, 3.1%), hairy cell leukemia (2 cases, 1.2%), marginal zone lymphoma (2 cases, 1.2%), follicular lymphoma (1 case, 0.6%), mantle cell lymphoma (1 case, 0.6%), low-grade B-cell lymphoma, unclassified (3 cases, 1.8%), plasma cell myeloma (3 cases, 1.8%), monoclonal gammopathy of undetermined significance (MGUS) (5 cases, 3.1%), diffuse large B-cell lymphoma (4 cases, 2.4%), high-grade B-cell lymphoma (1 case, 0.6%), and T-cell lymphoma (angioimmunoblastic T-cell lymphoma involved bone marrow) (1 cases, 0.6%). One case (0.6%) is mixed-phenotype acute leukemia (MPAL).

### 3.1. Interval between Peripheral Blood and Bone Marrow NGS

The interval between peripheral blood and bone marrow NGS ranged from 0 to 334 days with an average of 63 days. 80 cases had an interval of less than 30 days and 83 cases had an interval of more than 30 days.

### 3.2. Correlation between Peripheral Blood and Bone Marrow Mutational Analysis by NGS

150 out of 163 cases (92.0%) showed complete or partial concordance between both peripheral blood and bone marrow NGS. Complete concordance was seen in 124 cases (76.1%), and partial concordance was seen in 26 cases (15.9%). Discordance was seen in 13 cases (8.0%); of these, all were due to abnormal genes detected in one testing modality but none detected in the other modality. The correlation between peripheral blood and bone marrow NGS showed good concordance with a kappa coefficient of 0.794 (kappa standard error 0.054) and *P* value for testing kappa <0.0001 (Table 1).

When including only cases with an interval between peripheral blood and bone marrow NGS of less than 30 days, complete or partial concordance was seen in 72 out of 80 cases (90.0%). Cases with an interval of less than 30 days demonstrated no significant difference from cases with more than a 30-day interval (Table 2).

### 3.3. Correlation between Peripheral Blood and Bone Marrow NGS by Disease

We assessed the concordance across the diagnostic group and the results are shown in Table 3. Nonneoplastic abnormal blood count showed concordance in 87.0% with a kappa coefficient of 0.743 and a kappa standard error = 0.089 (strength of agreement = good). Myeloid neoplasm (including MPAL) showed concordance results in 98.7% with a kappa coefficient of 0.916 and a kappa standard error = 0.083 (strength of agreement = very good). Lymphoid neoplasm showed concordance results in 83.9% with a kappa coefficient of 0.599 and a kappa standard error = 0.157 (strength of agreement = moderate).

When stratified by diagnosis, all of the cases within many diagnosis groups showed complete or partial concordance between peripheral blood and bone marrow NGS. These included myelodysplastic syndrome (MDS) (21 cases, 100%), myeloproliferative neoplasm (MPN) (21 cases, 100%), myelodysplastic syndrome/myeloproliferative neoplasm (MDS/MPN) (11 cases, 100%), CLL (5 cases, 100%), hairy cell leukemia (2 cases, 100%), myeloma (3 cases, 100%), mastocytosis (1 case, 100%), and mixed-phenotype acute leukemia (MPAL) (1 case, 100%). Discordance was seen in 7 nonneoplastic abnormal blood count cases, 4 mature lymphoid neoplasm cases, 1 acute myeloid leukemia (AML) case, and 1 B-lymphoblastic leukemia (BALL) case (Table S1).

Nonneoplastic abnormal blood count cases showed complete or partial concordance in the majority of cases (87.0%). In six out of seven discordant cases, abnormal genes were detected in bone marrow NGS but not in peripheral blood NGS, while in one case, an abnormal gene was found in peripheral blood NGS but not in bone marrow NGS.

Myeloid neoplasm showed complete or partial concordance in 77/78 cases (98.7%). Complete concordance was seen in 59/78 cases (75.6%) and partial concordance was seen in 18/78 cases (23.1%). Discordance was found in 1 case (1.3%), in which the diagnosis was AML.

Lymphoid neoplasm showed complete or partial concordance in 26 out of 31 cases (83.9%). Complete concordance was seen in 21 out of 31 cases (67.7%), and partial concordance was seen in 5 out of 31 cases (16.1%). Discordance was observed in 5 cases (16.1%), which included one BALL, two DLBCL involving marrow, one marginal zone lymphoma involving marrow, and one MGUS.

## 4. Discussion

Our study demonstrated a high degree of concordance between mutational analysis by NGS on peripheral blood and bone marrow. The concordance rate that we observed was similar to a previous study limited to MDS patients performed by Mohamedali et al. [8, 9] We found that the concordance of peripheral blood and bone marrow NGS showed no significant differences by the time interval in between the acquisition of the samples; results on the samples collected less than 30 days versus upto a year.

Peripheral blood NGS showed a high degree of concordance with bone marrow NGS in various settings. In nonneoplastic abnormal blood count patients, concordant results were found in the majority of cases (87.0%). Discordant results were seen in 7 patients; the discordant genes were *DNMT3A* (3 patients), *ASXL1* (2 patients), *CEBPA* (1 patient), *BCORL1* (1 patient), and *TP53* (1 patient). One possible explanation is that *DNMT3A* and *ASXL1* known to represent upto two-thirds of the clonal hematopoiesis genes were found in low allele frequency in the bone marrow but not in the peripheral blood.

Regarding myeloid neoplasms, we found that MDS, MPN, and MDS/MPN patients (total of 53 patients) showed a 100% correlation of NGS results obtained from peripheral blood and bone marrow, similar to previous studies in MDS patients [8, 9]. In acute leukemia patients, concordance was seen in 25 out of 27 cases (92.59%). High concordance was observed in myeloid and biphenotypic leukemia (95.83%) and lower concordance was seen in BALL cases; this result is expected as patients with BALL occasionally presented with no peripheral blasts.

Overall, these findings show that peripheral blood NGS shows a high concordance with bone marrow NGS, particularly when evaluating myeloid neoplasms. Conversely, concordance may be partial in the setting of diseases such as high grade MDS with bone marrow blasts and disproportionately lower blasts % in the peripheral blood.

Lymphoid neoplasms showed concordance in 83.9% of cases. This degree of concordance is lower than that seen in myeloid neoplasms. This finding is expected due to the lack of circulating neoplastic cells in many lymphoid neoplasms. In lymphoid neoplasm with peripheral circulating neoplastic cells such as CLL and hairy cell leukemia, the peripheral blood and bone marrow showed concordance in 100% of the cases.

The discordant results were observed in a “bidirectional” fashion as demonstrated in a previous study [9]. The majority of discordant cases detect abnormal genes in the bone marrow specimen not in peripheral blood. Conversely, in two out of thirteen discordant cases, genes were only detected in peripheral blood NGS, while NGS obtained from bone marrow showed no mutations. This discordance may be due to suboptimal bone marrow sampling, e.g., subcortical marrow biopsy, aparticulate bone marrow aspiration, and marrow fibrosis. Therefore, peripheral blood NGS can provide additional diagnostic value in cases that have limited diagnostic bone marrow material.

Our findings can be immensely helpful in clinical practice, especially when the diagnostic material for bone marrow studies is aparticulate and hence insufficient for ancillary studies. In addition, our study further validates the role of periodic peripheral blood NGS assays for screening patients with peripheral blood cytopenias as a guide to the overall bone marrow status, especially in patients with clonal cytopenias of undetermined significance (CCUS) or low-grade MDS. However, it is important to streamline the approval process for peripheral blood NGS studies only to be used in the appropriate setting as the assay may detect mutations of undetermined significance and germline mutations that are not pathogenic and hence of no clinical significance leading to unwanted confusion.

We acknowledge that there are some limitations to our study. The concordance between the two samples greatly depends on the distribution of the cells of interest in both the specimens. Therefore, peripheral blood NGS may not be a good tool to study the clonal architecture in clinical entities that are marrow-centric diseases like some lymphomas. The other limitation of this approach is in cases of ALL and high-grade MDS with increased blasts, where the blast % in the peripheral blood may not be representative of the bone marrow disease burden. One way to overcome this problem would be to perform flow cytometry on the peripheral blood before peripheral blood sequencing to increase the reliability of the peripheral blood NGS studies. One of the future directions includes studying paired posttreatment samples from patients who have undergone induction chemotherapy or bone marrow transplantation, especially since the bone marrow sampling is often hypocellular during this treatment period.

## 5. Conclusion

Mutational analysis by peripheral blood NGS showed significant concordance with bone marrow NGS. Myeloid neoplasms showed a very high concordance while slightly lower levels of concordance was seen in lymphoid neoplasms and nonneoplastic abnormal blood counts. Our findings demonstrate that peripheral blood NGS is a reliable tool for mutational analysis and can provide a less invasive method for screening and monitoring molecular profile in hematolymphoid conditions.

## Figures and Tables

**Table 1:** Correlation of peripheral blood NGS and bone marrow NGS.

Total cases	Count (*N* = 163)	Percentage (%)	Kappa	Standard error of kappa	*P* value
Concordance	150	92.0	0.794	0.054	<0.0001
Complete concordance	124	76.1			
Partial concordance	26	15.9			
Discordance	13	8.0			

**Table 2:** Correlation of peripheral blood NGS and bone marrow NGS.

Cases interval 30 days or less	Count (*N* = 80)	Percentage	Kappa	Standard error of kappa	*P* value
Concordance	72	90.0%	0.750	0.083	<0.0001
Complete concordance	59	73.7%			
Partial concordance	13	16.3%			
Discordance	8	10.0%			

Cases more than 30 days interval	Count (N = 83)	Percentage	Kappa	Standard error of kappa	*P* value

Concordance	78	94.0%	0.839	0.069	<0.0001
Complete concordance	65	78.3%			
Partial concordance	13	15.7%			
Discordance	5	6.0%			

**Table 3:** Correlation of NGS result between peripheral blood and bone marrow NGS grouped by diagnostic groups.

Diagnoses	Count	Percentage (%)	Kappa	Standard error of kappa	*P* value
Nonneoplastic abnormal blood count (*n* = 54)					
**Concordance**	47	87.0	0.743	0.089	<0.0001
Complete concordance	44	81.4			
Partial concordance	3	5.6			
**Discordance**	7	13.0			
**Myeloid neoplasms (n** **=** **78)**					
**Concordance**	77	98.7	0.916	0.083	<0.0001
Complete concordance	59	75.6			
Partial concordance	18	23.1			
**Discordance**	1	1.3			
**Lymphoid neoplasms (n** **=** **31)**					
**Concordance**	26	83.9	0.599	0.157	0.00014
Complete concordance	21	67.7			
Partial concordance	5	16.1			
**Discordance**	5	16.1			

## Data Availability

The data are available from the corresponding author on request.

## Supplementary Materials

Supplementary Materials Table S1: correlation of NGS result between peripheral blood and bone marrow NGS grouped by diagnoses.
