# Supplementary material for: Concordance of Peripheral Blood and Bone Marrow Next-Generation Sequencing in Hematologic Neoplasms
Source: Adv Hematol. 2022 Mar 26;2022:8091746. doi: 10.1155/2022/8091746 (PMC8976630; doi:10.1155/2022/8091746)
Supplement: Supplementary Materials — Table S1: correlation of NGS result between peripheral blood and bone marrow NGS grouped by diagnoses. [file 8091746.f1.docx]

***Table S1:*** *Correlation of NGS result between peripheral blood and bone marrow NGS grouped by diagnoses*

| **Diagnoses** | **Count** | **Percentage** |  | **Diagnoses** | **Count** | **Percentage** |
| --- | --- | --- | --- | --- | --- | --- |
| **Non-neoplastic abnormal blood count (n=54)** | | |  | **MPAL (n=1)** | | |
| **Concordance** | 47 | 87.0% |  | **Concordance** | **1** | **100.0%** |
| Complete concordance | 44 | 81.4% |  | Complete concordance | 0 | 0.0% |
| Partial concordance | 3 | 5.6% |  | Partial concordance | 1 | 0.0% |
| **Discordance** | 7 | 13.0% |  | **Discordance** | **0** | **0.0%** |
| **MDS (n=21)** | | |  | **Low-grade B-cell lymphoma (n=14)** | | |
| **Concordance** | **21** | **100.0%** |  | **Concordance** | **13** | **92.9%** |
| Complete concordance | 16 | 76.2% |  | Complete concordance | 11 | 78.6% |
| Partial concordance | 5 | 23.8% |  | Partial concordance | 2 | 14.3% |
| **Discordance** | **0** | **0.0%** |  | **Discordance** | **1** | **7.1%** |
| **MPN (n=21)** | | |  | **High-grade B-cell lymphoma / DLBCL (n=5)** | | |
| **Concordance** | **21** | **100.0%** |  | **Concordance** | **3** | **60.0%** |
| Complete concordance | 17 | 81.0% |  | Complete concordance | 3 | 60.0% |
| Partial concordance | 4 | 19.0% |  | Partial concordance | 0 | 0.0% |
| **Discordance** | **0** | **0.0%** |  | **Discordance** | **2** | **40.0%** |
| **MDS/MPN (n=11)** | | |  | **Plasma cell dyscrasia (n=8)** | | |
| **Concordance** | **11** | **100.0%** |  | **Concordance** | **7** | **87.5%** |
| Complete concordance | 8 | 72.7% |  | Complete concordance | 5 | 62.5% |
| Partial concordance | 3 | 27.3% |  | Partial concordance | 2 | 25.0% |
| **Discordance** | **0** | **0.0%** |  | **Discordance** | **1** | **12.5%** |
| **AML (n=23)** | | |  | **T-cell lymphoma (n=1)** | | |
| **Concordance** | **22** | **95.7%** |  | **Concordance** | **1** | **100.0%** |
| Complete concordance | 18 | 78.3% |  | Complete concordance | 1 | 100.0% |
| Partial concordance | 4 | 17.4% |  | Partial concordance | 0 | 0.0% |
| **Discordance** | **1** | **4.3%** |  | **Discordance** | **0** | **0.0%** |
| **B-ALL (n=3)** | | |  | **Mastocytosis (n=1)** | | |
| **Concordance** | **2** | **66.7%** |  | **Concordance** | **1** | **100.0%** |
| Complete concordance | 1 | 33.3% |  | Complete concordance | 0 | 0.0% |
| Partial concordance | 1 | 33.3% |  | Partial concordance | 1 | 100.0% |
| **Discordance** | **1** | **33.3%** |  | **Discordance** | **0** | **0.0%** |
